# Newly established gastrointestinal cancer cell lines retain the genomic and immunophenotypic landscape of their parental cancers

**DOI:** 10.1038/s41598-020-74797-0

**Published:** 2020-10-21

**Authors:** Daniela Hirsch, Steffen Seyfried, Tobias Staib, David Fiedler, Christian Sauer, Thomas Ried, Stephanie Witt, Felix Rueckert, Timo Gaiser

**Affiliations:** 1 Institute of Pathology, University Medical Center Mannheim, Medical Faculty Mannheim, Heidelberg University, Theodor-Kutzer-Ufer 1-3, 68167 Mannheim, Germany; 2 Section of Cancer Genomics, Genetics Branch, Center for Cancer Research, National Cancer Institute, National Institutes of Health, Bethesda, MD USA; 3 Department of Surgery, University Medical Center Mannheim, Medical Faculty Mannheim, Heidelberg University, Mannheim, Germany; 4 Molecular Genetic Laboratory and Biobank, Department of Genetic Epidemiology in Psychiatry, Central Institute for Mental Health (CIMH), Medical Faculty Mannheim, Heidelberg University, Mannheim, Germany

**Keywords:** Cancer genetics, Cancer genomics, Cancer models, Gastrointestinal cancer

## Abstract

Human cancer cell lines are frequently used as model systems to study molecular mechanisms and genetic changes in cancer. However, the model is repeatedly criticized for its lack of proximity to original patient tumors. Therefore, understanding to what extent cell lines cultured under artificial conditions reflect the phenotypic and genomic profiles of their corresponding parental tumors is crucial when analyzing their biological properties. To directly compare molecular alterations between patient tumors and derived cell lines, we have established new cancer cell lines from four patients with gastrointestinal tumors. Tumor entities comprised esophageal cancer, colon cancer, rectal cancer and pancreatic cancer. Phenotype and genotype of both patient tumors and derived low-passage cell lines were characterized by immunohistochemistry (22 different antibodies), array-based comparative genomic hybridization and targeted next generation sequencing (48-gene panel). The immunophenotype was highly consistent between patient tumors and derived cell lines; the expression of most markers in cell lines was concordant with the respective parental tumor and characteristic for the respective tumor entities in general. The chromosomal aberration patterns of the parental tumors were largely maintained in the cell lines and the distribution of gains and losses was typical for the respective cancer entity, despite a few distinct differences. Cancer gene mutations (e.g., *KRAS*, *TP53*) and microsatellite status were also preserved in the respective cell line derivates. In conclusion, the four examined newly established cell lines exhibited a phenotype and genotype closely recapitulating their parental tumor. Hence, newly established cancer cell lines may be useful models for further pharmacogenomic studies.

## Introduction

Cancer is a heterogeneous disease in terms of both phenotype and genotype. Various types of tumors with different histomorphologies, genetic and epigenetic alterations, and clinical courses exist^1^. Since the establishment of the cervical carcinoma cell line HeLa in 1951, human cancer cell lines have widely been used as model systems to study molecular mechanisms and genetic changes in cancer^2,3^. The advantages of established “immortal” cell lines as two-dimensional in vitro cancer models are obvious: easy to maintain and to (molecularly) manipulate, fast growing (short doubling time), cheap, broadly and easily available, potentially animal protecting and represent a source for unlimited production of specific tumor DNA, RNA and protein^4^. Cancer cell lines represent pure populations of cancer cells, facilitating the detection of molecular genetic changes, in particular homozygous deletions. However, the cell line model is repeatedly criticized due to its limited reflection of in vivo solid tumors which are not simply pure cancer cells but include a variety of other cell types such as inflammatory cells, stromal cells and blood vessels. Furthermore, cross-contamination of cell lines, microbial contamination, misidentification of the tissue of origin and over-passaging are critical issues, in particular with respect to continuous long-term cell lines^5,6^.

Large-scale comparisons of the genomic profiles of established cell lines and primary patient tumors from The Cancer Genome Atlas (TCGA) project showed for colon cancer and ovarian high-grade serous cancer that overall established cell lines resemble the genomic changes and molecular subtypes observed in patient tumors fairly well, despite some incongruence^7–9^. Interestingly, more frequently used cell lines often had less genomic similarities to primary tumors than rarely used cell lines^9^. Given that tumorigenesis can be viewed as an evolutionary process, this illustrates that cell lines can apparently diverge from their primary tumor counterparts over time during pro-longed culture in vitro. How fast and to which extent this genomic evolution occurs is influenced by the inherent genomic instability of the tumor cells and the selective pressures such as culture conditions (as opposed to host microenvironment) they are subject to. For instance, Mouradov et al. identified an overrepresentation of *CTNNB1* mutations and microsatellite instability in established colon cancer cell lines compared to patient tumors^8^. These genomic alterations might have been acquired or selected for in culture or might eventually be related to the preferential outgrowth of particular genotypes, given that establishment of new cell lines remains a challenge and only about 10–15% of CRCs give rise to cell lines^8^. Ince et al. reported that improved culture conditions produce cell lines with genotypes and phenotypes more closely resembling primary tumors^10^. Hence, a culture context dependent genomic evolution when propagating tumor cells outside their host environment cannot be denied^11,12^. This is corroborated by more recent findings from Ben-Uri et al., showing that during patient-derived xenograft (PDX) passaging particular copy number alterations (CNAs) were acquired that differed from the ones seen in patients, while certain CNAs characteristic for and recurrently observed in patient tumors gradually disappeared during PDX passaging such as the gain of chromosome arm 8q (harbors the *MYC* oncogene on 8q24.3) in breast cancers^13^. Moreover, considerable genomic heterogeneity has been identified between established cancer cell lines cultured independently in different laboratories, indicating ongoing genomic evolution^14,15^. Concordantly, data from single cell cloning showed single cell derived clones of established CRC cell lines select for but cannot fully maintain the genotype of the parental cell population^16^. All these findings highlight the critical role of culture conditions, prolonged passaging and the degree of genomic instability in shaping the evolution of cancer cell lines genomes and phenotypes away from patient tumors. Furthermore, this underlines the need for the establishment of new patient-derived models such as patient-derived cell lines (PDCLs), PDXs or patient-derived organoids (PDOs), which may better reflect the biologic properties of the tumors they were derived from. Considerably large biobanks of PDOs have been established for several tumor entities, leadingly by Clevers et al., and data comparing phenotype and genotype of these PDOs with corresponding patient tumors suggest a high overlap^17–21^. Establishment of new PDCL remains more difficult and hence data are sparser. Sample numbers of successful outgrowths are usually small, and most studies report the establishment of one or a few new cell lines, often along with some molecular or functional characterization of the newly generated cell lines. However, a direct comparison with the patient tumor from which the cell line was derived from is only rarely provided. We consider a thorough molecular characterization of new patient-derived models along with the tumors they were derived from crucial since the genotype can directly influence transcriptional changes and is correlated with drug response (e.g., anti-HER2 treatment in HER2-amplified cancers of breast, gastroesophageal junction and colorectum). To address challenges with current cancer cell lines, such as the lack of data on the tumors of origin and cancer subtypes that are missing or poorly represented, the Human Cancer Models Initiative (HCMI) has been established^22^. The HCMI is an international effort to generate, genomically characterize and annotate the new cancer cell models as a resource for the scientific community.

Here, we report the establishment of four new PDCLs from different tumors of the digestive system including esophageal cancer, colon cancer, rectal cancer and pancreatic ductal adenocarcinoma (PDAC). We performed a direct side-by-side comparison with the respective parental tumors to determine to what extent the newly established low-passage cell lines reflect the phenotypic, genomic and genetic characteristics of the tumor from which they have been derived. To this end, both parental tumors and corresponding derived cell lines were analyzed by immunohistochemistry, array comparative hybridization (aCGH) and targeted next generation sequencing (TruSeq Amplicon 48-gene panel). This allowed us to assess how faithfully newly established PDCLs model their tumors of origin.

## Material and methods

### Patients and tissue samples

Fresh tumor samples for cell culture were acquired from patients who underwent surgery for a tumor of the digestive system at University Medical Center Mannheim, Germany. Samples included in this study were taken from an esophageal carcinoma (liver metastasis), a colonic adenocarcinoma, a rectal adenocarcinoma and a pancreatic ductal adenocarcinoma, diagnosed between 2012 and 2015. Corresponding formalin-fixed paraffin-embedded (FFPE) parental tumor tissue was obtained from the archive of the Institute of Pathology, University Medical Center Mannheim, Germany. In addition to clinical diagnosis and the pathology report, the diagnosis was histologically confirmed by two pathologists (TG, DH). Tumor diagnosis and staging was performed according to the WHO classification and the American Joint Committee on Cancer (AJCC)/Union for International Cancer Control (UICC) staging system^23,24^. All patients gave informed consent prior to surgery, and the study was approved by the local ethics committee of the Medical Faculty Mannheim of Heidelberg University (2012-293N-MA) and by the institutional review board of the National Institutes of Health (OHSRP #13221/MTA #41443). All experiments were performed in accordance with relevant guidelines and regulations.

### Cell culture procedure

To establish cell lines, fresh tumor tissues were processed by the outgrowth method as described previously^25^. Briefly, fresh tumor tissues were cleaned from surrounding connective tissue and necrotic and/or hemorrhagic areas, and subsequently finely minced into cubes of approximately 1 mm^3^ using two scalpels. Neither enzymatic nor mechanic dissociation of the tumor cells was performed. Basal culture media were as follows: Dulbecco's Modified Eagle Medium (DMEM-high glucose; cat # D5796-500ML, Sigma-Aldrich, St. Louis, MO, USA; for liver metastasis of esophageal carcinoma and for colorectal adenocarcinomas, respectively) or DMEM/keratinocyte serum free medium mixed at a ratio of 2 to 1 (KSFM; cat # 17005042, Gibco, Thermo Fisher Scientific, Waltham, MA, USA; for PDAC). Basal media were supplemented with fetal bovine serum (20% v/v; cat # F9665-500ML, Sigma), penicillin (100 U/ml; cat # 11074440001, Sigma) and gentamicin (2.5 mg/ml). The cells were cultured at 37 °C in a humidified atmosphere of 5% CO_2_ in air, and the medium was replaced every third day. All experiments for this study were performed on cell lines between the first and fourth passage. Cell lines tested negative for mycoplasma contamination and were authenticated using short-tandem repeat (STR) profiling.

### STR profiling

STR profiling was done according to the recommendations of the Standard for human cell line authentication established by the American Type Culture Collection Standards Development Organization Workgroup^26^. The following loci were used: amelogenin, CSF1PO, D5S818, D7S820, D13S317, D16S359, TH01, TPOX, and vWA. For cell line authentication, the Cellosaurus STR Similarity Search Tool (CLASTR; version 1.4.3) was used^27^.

### Preparation of cell blocks

Cultured adherent cells were dissociated using Trypsin–EDTA (0.25%) (cat # 25200056, Gibco). Cell suspensions of cultured cells were spun down at 300*g* for 10 min and supernatant (culture medium) was discarded. Cell pellets were re-suspended 1:1 in 3% (w/v) agar agar (A1296-100G, Sigma). Once cooled down and solidified, the cells agar mixture was placed in a tissue embedding cassette and subsequently formalin-fixed and paraffin-embedded for further processing.

### Immunohistochemical staining

Two to three µm sections from tissue blocks (parental tumors) or cell blocks (derived cell lines) were mounted on Superfrost Plus microscope slides (Thermo Fisher Scientific, Waltham, MA, USA). After deparaffinization and re-hydration, heat-induced antigen retrieval (HIAR) was performed in Novocastra Epitope Retrieval Solution pH 9 or pH 6 (both Leica Biosystems, Wetzlar, Germany) in a water bath at 95 °C for 20 min (pH 9) or 40 min (pH 6), respectively, followed by incubation of sections with primary antibodies for 30 min at room temperature (see Supplementary Table S1 for primary antibodies and respective HIAR conditions). Detection was done using the EnVision Detection System, Peroxidase/DAB, Rabbit/Mouse (cat # K5007, Dako). All stainings were validated by internal and/or external positive controls as well as negative control specimens. Immunohistochemical stains of original tumors and derived cell lines were quantified using the H score method, which takes into account the percentage of positive tumor cells (0–100%) and their staining intensity (0 for negative, 1+ for weakly positive, 2+ for moderately positive, 3+ for strongly positive)^28,29^. The parameters were determined by visual assessment of two pathologists (DH, TG). The H score was calculated according to the following formula, ranging from 0 to 300:

$$H score=1 \times \left(\% of 1+ cells\right) + 2 \times \left(\% of 2+ cells\right) + 3 \times (\% of 3+ cells)$$

The relationship of H scores of parental tumors and derived cell lines was evaluated using Pearson’s correlation. Microscopy images were acquired with a digital microscope and scanner M8 (PreciPoint GmbH, Freising, Germany).

### DNA isolation

DNA was isolated from fresh or frozen cells using the DNeasy Blood and Tissue Mini Kit (Qiagen, Hilden, Germany). From FFPE tumor tissue (two spatially separated/distinct tumor regions per case), DNA isolation was done as described previously^30^. All samples were evaluated based on 3-µm hematoxylin eosin (H&E) stained sections and the tumor regions for DNA isolation were marked for macrodissection. Tumor cell contents of the parental tumors were 50% (patient 1 & 2), 40% (patient 3) and 20% (patient 4), respectively. DNA concentration and purity were measured by a NanoDrop 1000 Spectrophotometer (NanoDrop products, Wilmington, DE, USA). In addition, double-stranded DNA was quantified by a Qubit 3.0 fluorometer (Life Technologies, Thermo Fisher Scientific, Waltham, MA, USA) using the Qubit dsDNA BR (Broad Range) Assay Kit (Life Technologies). DNA integrity was evaluated on 1% agarose gels stained with Gel-Red (Biotium, Hayward, CA, USA). BIOMED-2 control gene PCR assay of differently sized amplicons (targeting 600, 400, 300, 200 and 100 bp fragments of the human genes *AFF1*, *PLZF*, *RAG1* and *TBXAS1*, respectively) was performed to visualize DNA fragment size distribution and to test amplifiability and integrity of DNA samples^31^. In addition, amplifiability of the isolated FFPE DNA was assessed by a qPCR assay (Illumina FFPE QC Kit for TruSeq Amplicon—Cancer Panel, Illumina, San Diego, CA, USA) and delta Ct values were determined against the provided control DNA (ACD1) and as additional reference against human genomic DNA (Promega, Madison, WI, USA).

### Array-based comparative genomic hybridization

DNA isolated from fresh/frozen cells was enzymatically labeled using the SureTag Labeling System (Agilent, Santa Clara, CA, USA) and DNA from FFPE tumor tissue was labeled using the Genomic DNA ULS Labeling Kit (Agilent) as described previously^32^. Sex-matched human genomic DNA (Promega, Madison, WI, USA) was used as reference. Labeled DNA was hybridized to SurePrint G3 CGH 8X60K microarrays (Agilent). Microarrays were scanned with microarray scanner G2565BA (Agilent) and microarray images were processed by Feature Extraction software version 10.7.3.1 (Agilent). Data were visualized and analyzed using Nexus Copy Number software version 9.0 (BioDiscovery, Inc., El Segundo, CA, USA). Clustering was done using the average linkage hierarchical clustering algorithm. The percentage of genome detected concordantly as gain, loss or neutral between parental tumor and derived cell line was calculated (overlap/overlapping calls).

### Targeted next generation sequencing

Libraries of fresh and FFPE isolated DNA (250 ng) were prepared using the TruSeq Amplicon—Cancer Panel (Illumina). The TruSeq Amplicon—Cancer Panel spans mutational hotspots in > 35 kilobases (kb) of target genomic sequence in the following genes: *ABL1, AKT1, ALK, APC, ATM, BRAF, CDH1, CDKN2A, CSF1R, CTNNB1, EGFR, ERBB2, ERBB4, FBXW7, FGFR1, FGFR2, FGFR3, FLT3, GNA11, GNAQ, GNAS, HNF1A, HRAS, IDH1, JAK2, JAK3, KDR, KIT, KRAS, MET, MLH1, MPL, NOTCH1, NPM1, NRAS, PDGFRA, PIK3CA, PTEN, PTPN11, RB1, RET, SMAD4, SMARCB1, SMO, SRC, STK11, TP53, VHL*. The PhiX control library (Illumina) was spiked in each run at a final concentration of 10 pM to estimate the sequencing error rate. The pooled libraries were paired-end (2 × 151) sequenced on a MiSeq instrument (Illumina). The mean read depth for targeted regions (mean coverage) was ~ 1400×. Alignment and variant calling were done using the BaseSpace TruSeq Amplicon App, Version 3.0.0 (Illumina) using the somatic variant caller. Annotation was done based on RefSeqGene (https://www.ncbi.nlm.nih.gov/refseq/rsg/) and variants were further evaluated based on dbSNP 147 (NCBI; https://www.ncbi.nlm.nih.gov/projects/SNP/), COSMIC database (Catalogue of Somatic Mutations in Cancer; https://cancer.sanger.ac.uk/cosmic) and ClinVar database^33–35^. Only non-synonymous variants were considered. Specific filtering criteria included (1) passed variant caller filters, (2) read depth ≥ 10 with a fraction of alternative reads ≥ 0.1, (3) ExAC frequency < 0.001%. Reads were visualized using the Integrative Genomics Viewer (IGV, Broad Institute)^36,37^.

## Results

### Establishment of primary cancer cell lines from patient tissues

**Figure 1:**
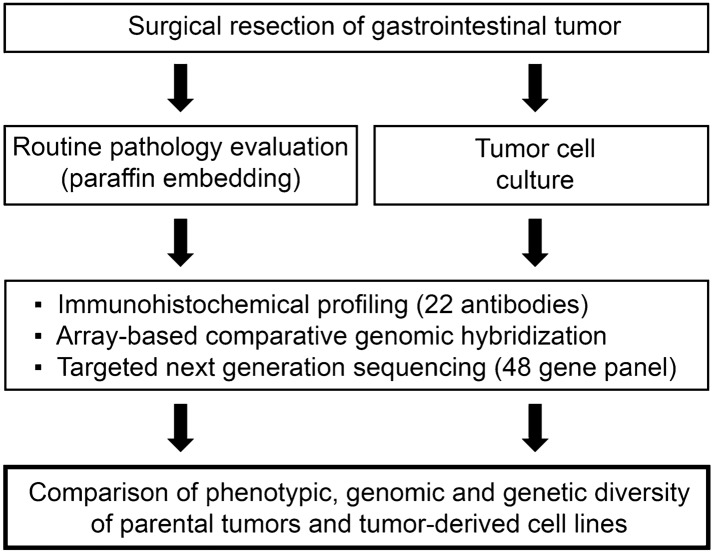
Overview of study design. Tumor resection specimens were submitted to the pathology department for diagnostic assessment, in parallel a small tumor sample was cultured according to the described procedure. Successful tumor cell cultures and respective parental tumors were molecularly characterized.

We attempted to characterize how well genomic and phenotypic characteristics of patient tumors are maintained in cell lines compared to their tumor of origin. Towards this goal, we aimed to generate primary cancer cell lines from different gastrointestinal carcinomas using patient specimens (Fig. 1).

We succeeded in establishing new primary cell lines from four patients comprising the following tumor entities: esophageal carcinoma, colon carcinoma, rectal carcinoma and pancreatic cancer (Table 1). The majority of primary tumors were locally advanced, and two patients had distant metastases. Three cell lines were established from the primary tumor, one cell line from a liver metastasis of the respective tumor. The original tumors showed a moderate to poor differentiation. All four newly established cell lines were maintained in serum-containing medium, and grew in an adherent, i.e., anchorage-dependent, manner. All cell lines were verified for absence of fibroblasts (as determined by microscopy and immunohistochemistry) and STR profiling was performed to proof individual identity of the cell lines and the derivation from the respective patient tumor (Supplementary Table S2). Additionally, freezing down and re-culturing after thawing was tested successfully for each cell line.

**Table 1:** Patient characteristics and clinico-pathologic data.

	Patient 1	Patient 2	Patient 3	Patient 4
Age at surgery (years)	46	69	65	64
Sex	Male	Male	Male	Male
Ethnicity	Caucasian	Caucasian	Caucasian	Caucasian
Tumor type	Esophageal adenocarcinoma	Colorectal adenocarcinoma	Colorectal adenocarcinoma	Pancreatic ductal adenocarcinoma
Tumor location	Liver (metastasis)	Rectum	Ascending colon	Head of pancreas
TNM stage	ypT0 ypN2 (6/47) L0 V0 Pn0 R0 ypM1 (HEP)	pT2 pN0(0/14) L0 V0 Pn0 R0	pT4b pN1a(1/13) L0 V0 Pn0 R0 pM1(LYM,PER)	pT3 pN1(3/20) L0 V0 Pn1 R0
Tumor grade	N/A	G2	G3	G3
Differentiation	Dedifferentiated	Moderate	Poor	Poor
In vivo growth pattern	Solid, partially necrotic	Tubulo-papillary, cribriform	Trabecular, solid, partially necrotic	Invasive ductal, partially necrotic
MMR status (IHC)	MLH1+, MSH2+, MSH6+, PMS2+	MLH1+, MSH2+, MSH6+, PMS2+	MLH1−, MSH2+, MSH6+, PMS2-	MLH1+, MSH2+, MSH6+, PMS2+
Cell line established from	Liver metastasis	Primary tumor	Primary tumor	Primary tumor
Cell line name	ECMA-1	MCC-38	MCC-60	MaPac-77
In vitro growth pattern	Adherent	Adherent	Adherent	Adherent
Doubling time	87 h	22 h	42 h	71 h
Cell culture passage used for analysis	P2	P4	P1	P1

*IHC* immunohistochemistry, *MMR* mismatch repair.

### Cell lines recapitulate some histotype-specific morphologic features

**Figure 2:**
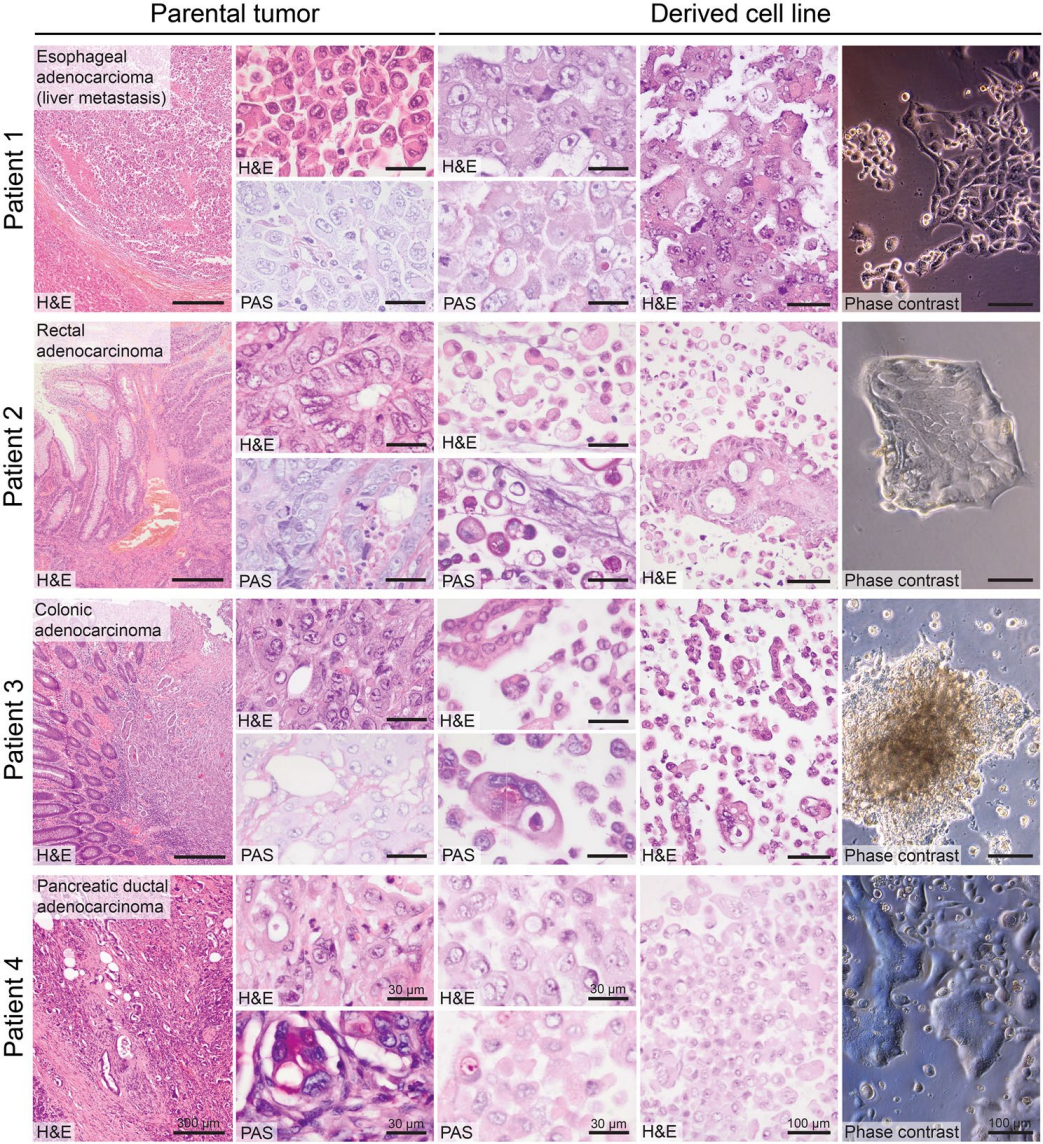
Morphology and growth patterns of parental tumors and tumor-derived cell lines. Shown are the histology images of parental tumors and derived cell lines along with the Periodic Acid Schiff (PAS) reaction for detection of mucus production and a phase contrast image of the tumor cell culture. Newly established cell lines mainly grew as solid, adherent monolayers with partial retention of histotype-specific features such as gland formation in adenocarcinomas (in particular patients 2 and 3) and focal mucin production as indicated by positive PAS reaction.

The newly established cell lines mainly grew as adherent, i.e., anchorage-dependent, monolayers in vitro, showing epithelial cell morphology and retaining some characteristic features of the respective parental tumors such as gland formation (Fig. 2). Interestingly, the two cell lines with colorectal origin did not only preserve some gland formation from the original tumor but also showed mucus production as indicated by the positive Periodic Acid Schiff (PAS) reaction. The pancreatic cancer derived cell line also displayed focal, subtle mucus production in the PAS reaction, which is in line with parental tumor histology. Both gland formation and mucin production are histotype-specific features of adenocarcinomas.

### Newly established cell lines retain the immunophenotypic expression profiles of their parental tumors

Next, we aimed to determine the similarity of the immunophenotype of parental tumors and derived cell lines. To this end, we applied a panel of 22 immunohistochemical markers. The panel included intermediate filaments (pan-cytokeratin, cytokeratin 7, cytokeratin 20, vimentin), adhesion molecules (E-cadherin, EpCAM), lineage-specific transcription factors (CDX2, SATB2, TTF-1), glycoproteins/mucins with differential expression across tumors (CA19-9, CEA, EMA/MUC1, MUC4), a neuroendocrine marker (chromogranin A), a growth factor receptor (HER2/neu), a cell cycle checkpoint protein (cyclin D1), and a proliferation marker (Ki-67). Furthermore, DNA mismatch repair proteins (MLH1, MSH2, MSH6, PMS2) and TP53 were included.

**Figure 3:**
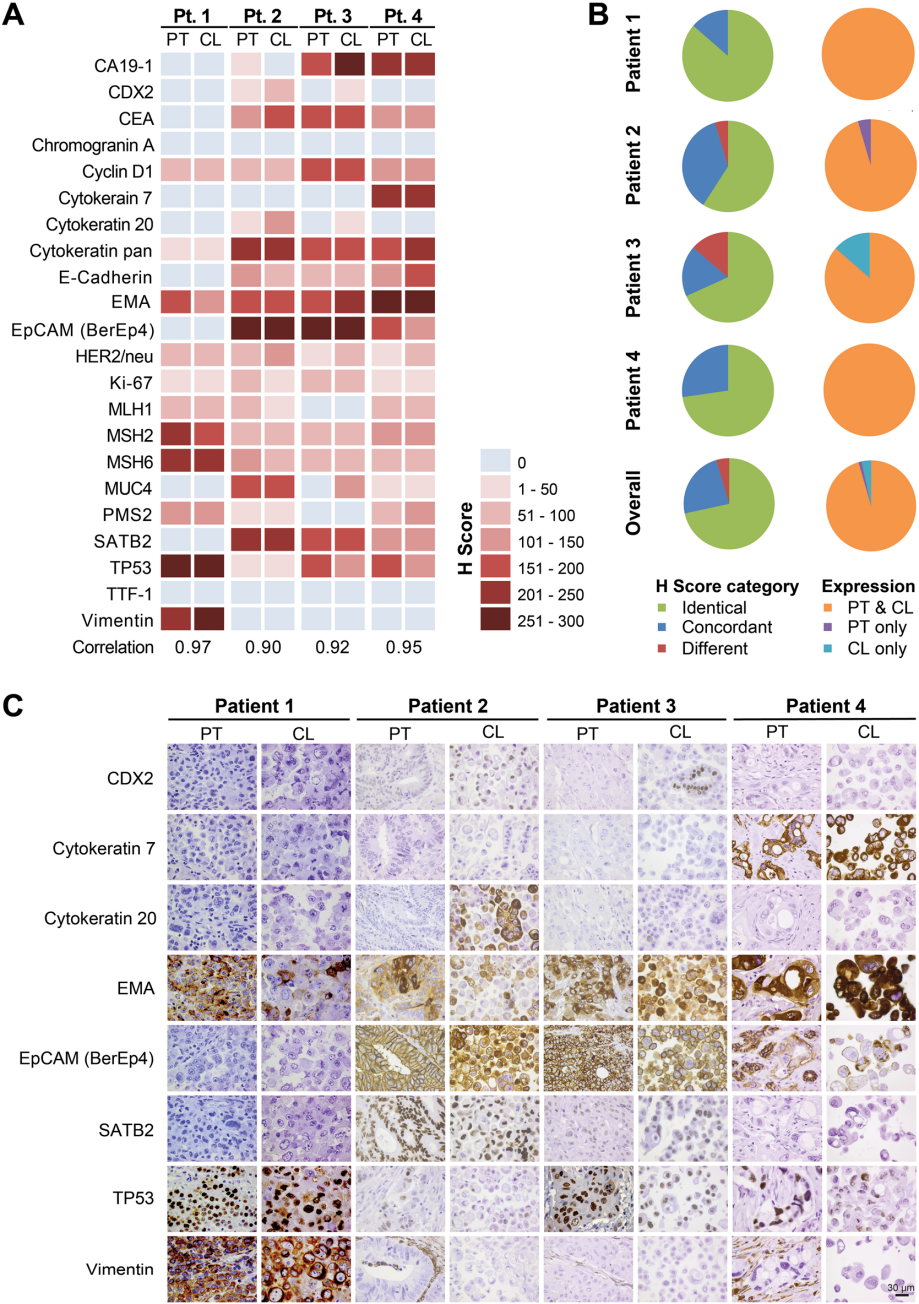
Immunohistochemical staining profiles of cell lines and corresponding parental tumors. (**A**) Heatmap of derived H scores showing strong similarities between cell lines and parental tumors. Correlation values represent the Pearson’s correlation coefficient r. (**B**) Pie charts summarizing distribution of shared and distinct H score categories and expression between tumors and derived cell lines. (**C**) Representative images of immunohistochemical stainings. *CL* cell line, *PT* parental tumor.

Overall, the newly established cell lines retained most of the immunophenotypic properties of the tumors they had been derived from, resulting in a strongly positive correlation as indicated by Pearson’s correlation coefficients r close to 1 ranging from 0.90 to 0.97 (Fig. 3, Supplementary Figs. S1 and S2). Spearman correlation showed similar results (Spearman Rho 0.88–0.99). Hence, newly established cell lines closely resemble the immunohistochemical staining profile of their tumors of origin. However, subtle differences could be observed with respect to staining degree and intensity as measured by the H score. Apart from markers with generally heterogeneous expression such as CA19-9 and MUC4, this particularly affected lineage-specific differentiation markers such as CDX2 along with lineage-specific cytokeratins such as CK20. However, none of the cell lines acquired the expression of a foreign lineage specific marker (e.g., TTF-1, a marker for lung and thyroid gland, was consistently negative in all cell lines). Also, no neuroendocrine differentiation was acquired in vitro based on chromogranin A. Interestingly, the colon cancer cell line and the parental primary tumor from patient 3 showed a loss of expression of DNA mismatch repair proteins MLH1 and PMS2 in the tumor cells, suggestive for microsatellite instability.

Taken together, our immunophenotypic profiling the newly derived cell lines revealed that the expression profiles of cell lines are largely shared with the tumor of origin and characteristic of the respective tumor types in general. These cell lines hence recapitulate overall the histologic and immunophenotypic features of the tumors they were derived from, despite certain differences.

### DNA copy number profiles of newly established cell lines and corresponding primary tumors are similar

**Figure 4:**
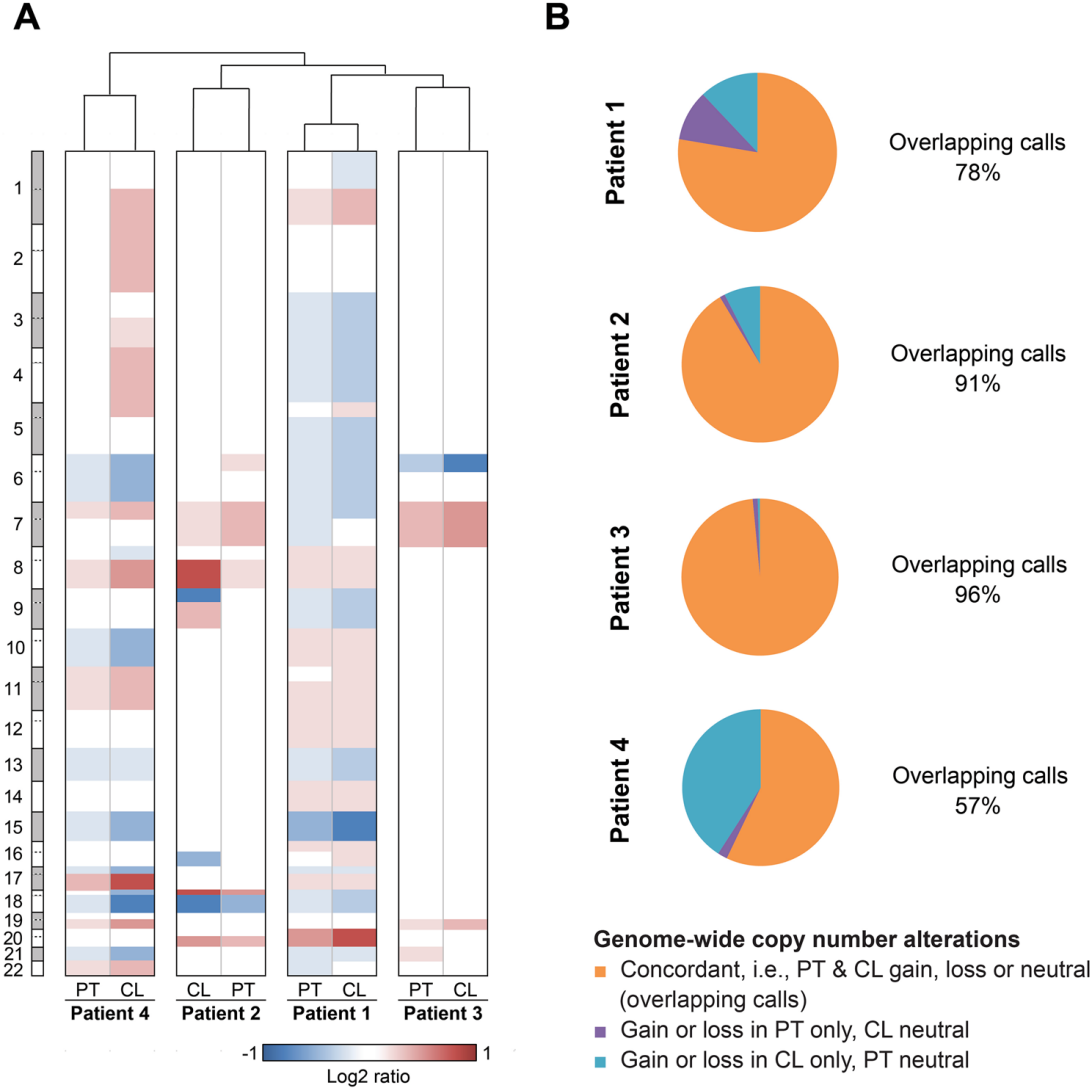
Copy number profiles of cell lines and corresponding patient tumors. (**A**) Unsupervised hierarchical clustering of genome-wide copy number alterations between matched tumors and tumor-derived cell lines, showing greater similarity of the cell lines to the respective parental tumors than to one another. Arm-level log2 ratios of chromosomes 1–22 are displayed as a heatmap where red indicates copy number gain and blue copy number loss. (**B**) For each patient the percentage of data points detected identically as gain, loss or neutral (overlap) along with discordant calls is displayed as pie charts. Overall copy number alteration detection was more sensitive in cell lines compared to original tumors as indicated by more calls in cell lines only. *CL* cell line, *PT* tumor.

To compare genome-wide DNA copy number changes of newly established cell lines and corresponding primary tumors, we performed aCGH analyses. The copy number profiles showed a non-random distribution of gains and losses that was characteristic for the respective tumor entities and similar, though not identical between parental tumors and tumor derived cell lines (Fig. 4, Supplementary Fig. S3). Parental tumor and the corresponding derived cell line always clustered together using the average linkage algorithm. The joint clustering of parental tumor and corresponding cell line remained robust when adding additional parental tumor samples (Supplementary Fig. S4). The amplitude of the observed gains and losses was generally higher in the cell lines compared to parental tumors due to the lack of contaminating non-malignant, i.e., immune and stromal, cells. As expected, the microsatellite instable tumor from patient 3 harbored only very few copy number alterations, largely confined to a loss of chromosome arm 6p and a gain of chromosome 7.

### Mutations in key genes are preserved in patient-derived cell lines

**Figure 5:**
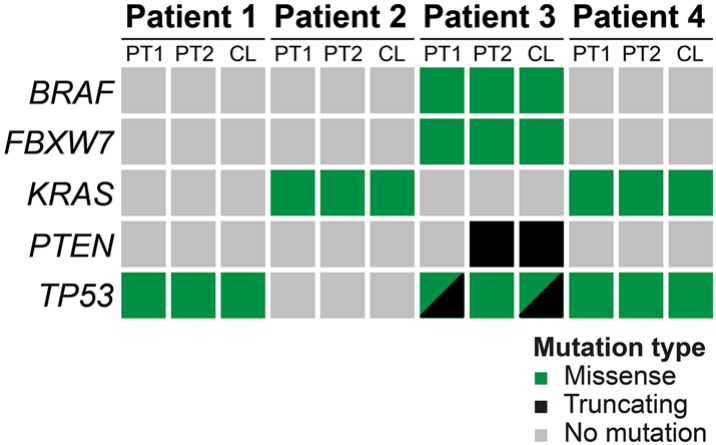
“Oncoprint” showing the key mutations shared by parental tumors and derived cell lines. For each patient, two spatially distinct parental tumor samples (PT1, PT2) and the derived cell line (CL) were sequenced. Mutations in key driver genes displayed little heterogeneity and were mostly shared by the parental tumor samples and derived cell line.

To determine shared and distinct key driver mutations, patient tumors and derived cell lines were sequenced with the TruSeq Amplicon Cancer Panel comprising hotspot mutation regions of 48 known cancer-related genes (see “Material and methods” section for gene list). For each patient, we analyzed mutations in two spatially distinct regions (two different FFPE blocks with tumor) to account for potential spatial heterogeneity. Mutations in key driver genes such as *TP53* and *KRAS* were consistently present in both primary tumor samples and the derived cell line (Fig. 5, Supplementary Table S3). However, we could also detect very few mutations, e.g., in *PTEN*, that were only detected in one of primary tumor samples but shared with the cell line. Not surprisingly, the microsatellite instable tumor from patient 3 had the highest number of mutations. Of note, the *TP53* mutation of cell lines from patients 1, 3 and 4 is also reflected in the immunohistochemical staining, which shows a mutation-typical staining pattern with strong nuclear accumulation of TP53 (Fig. 3).

## Discussion

Tumor cell lines are widely used model systems to study functional properties of human tumors including drug responses. Though cell lines overall seem to maintain genomic changes of the respective tumor entities, recent evidence indicates that cell lines undergo culture induced genomic evolution and selection in adaption to the artificial culture conditions over time^12^. Hence, on an individual basis, cultured cancer cell lines may not reflect the true properties of the original tumor they were derived from.

Here, we compared the genomic and phenotypic characteristics of newly generated, patient derived low-passage cell lines (passages 1–4) with their respective tumors of origin to assess in a direct manner how well patient tumor characteristics are maintained in culture. All newly established cell lines presented here grew in an epithelium-like pattern and largely retained the original tumor immunophenotype in vitro. The human epithelial nature was confirmed by cytokeratin expression in three out of four tumors (patients 2, 3 and 4, respectively)^38^. The tumor from patient 1 was diagnosed as a dedifferentiated carcinoma which had apparently undergone epithelial mesenchymal transition leading to the loss of keratin expression and other adhesion molecules such as e-cadherin while expression of vimentin was acquired. Maintained EMA expression is a hint for its epithelial origin^39^. Although overall derived cell lines largely maintained the immunophenotypic characteristics of the respective parental tumors, few discordantly expressed markers were observed when looking at an individual case basis. Features of intestinal differentiation as indicated by CDX2 and CK20 positivity were enhanced or re-acquired in the cell line while not or only weakly being expressed in the parental tumor (patients 2 and 3)^40,41^. SATB2, a newer marker for colonic differentiation, was more robust and consistently expressed in both parental CRCs and corresponding derived cell lines, underlining the diagnostic superiority for this marker compared to CDX2^42,43^.

Comparative analysis of chromosomal aneuploidy patterns by aCGH revealed generally consistent gain and loss patterns of parental tumors and derived cell line, reflective of the typical aneuploidy distributions observed in the respective tumor entities^44,45^. Clustering (average linkage algorithm) of copy number data separated microsatellite stable tumors from the microsatellite unstable CRC while paired tumor and cell line samples clustered together, demonstrating the similarity of their respective chromosomal aberration patterns. However, despite the overall similarity of the CNA patterns between parental tumor and corresponding cell lines, certain differences could be observed. Overall, signal to noise ratios tended to be lower in the parental tumors compared to the cell lines. This lower sensitivity in copy number detection is owed to the dilution of tumor cell DNA by DNA from intermingled stromal and inflammatory cells. This is most obvious in PDAC, which usually (and also in our case) has an extensive stromal component and is also evident in the TCGA PDAC cohort^46^. In our study, the PDAC-derived cell line was mainly characterized by losses of chromosomes and chromosome arms 6, 8p, 17p (harbors *TP53*) and 18q (harbors *SMAD4*) along with gains of chromosome arms 8q (harbors the *MYC* gene) and 17q, consistent with previous reports^46,47^. However, only a fraction of 57% of the gains and losses in the cell line could be detected in the primary tumor, which had a tumor cell content of only 20%. The gain of 8q (harbors *MYC*), observable in the primary tumor and in the cell line, has been proposed as a metastatic driver in pancreatic ductal adenocarcinoma^48^. Patient 4 from our cohort indeed developed liver metastases within three months after surgery.

In contrast, the cell line derived from the tumor of patient 2 (rectal adenocarcinoma) showed two differences that cannot necessarily be explained by a higher detection limit in patient tumors. The primary tumor and the derived cell line were characterized by gains of 7 and 20 along with a loss of 18q, a CNA pattern typical for CRC^49,50^. However, a loss of 9p and gain of 9q (possibly due to isochromosome formation), which do not typically occur in colorectal cancer, were solely detected in the cell line sample. One possible explanation for the discrepancy could be spatial intratumor copy number heterogeneity and the selective outgrowth of a tumor subclone that was underrepresented in the primary tumor^51,52^. Alternatively, this alteration could have been acquired during or induced by the culture process. Indeed, Greshock et al. have previously reported the loss of 9p, along with other copy number changes, as recurrent cell line-specific alterations^53^. A loss of 9p21.3 (*CDKN2A* locus) has also been described in the immortalization process of normal nasopharyngeal epithelial cells and may eventually confer a growth advantage in vitro under certain circumstances^54^. However, genomic alterations acquired during the culture process usually take time to be present in most of the cells (Li et al. report 63 population doublings).

The highest CNA burden was observed in the least differentiated tumor (esophageal cancer from patient 1). This is not surprising as CNA burden has previously been reported to be associated with more aggressive tumor behavior^55^. The microsatellite instable CRC and its derived cell line only displayed the lowest number of copy number alterations, which included a gain of chromosome 7, an event typically observed in CRC^49,50^.

As shown for colorectal cancer derived PDOs, mutations in known driver genes or genes of interest in the respective tumor entity (e.g., *KRAS* or *BRAF* in CRC) were invariably present in both primary tumor sample and derived cell line^18^. Only in the MSI cell line minor discrepancies for mutations in *PTEN* and *TP53* could be observed, likely attributable to the increased mutation rate as a consequence of MSI. We would like to emphasize that this did not affect mutations in driver genes such as *BRAF* or *KRAS*. This is in line with data from single cell subcloning of established CRC cell lines SW480 and HT29, which showed consistent mutations of *KRAS* and *BRAF*, respectively, across all established single cell clones—despite ongoing karyotype evolution/chromosomal instability^16^.

In conclusion, our low-passage cell lines closely recapitulated tumor-characteristic immunophenotypic profiles and maintained treatment-relevant genomic features including MSI status, key mutations of driver genes and tumor-type specific copy number alterations. Hence, this newly established cell line panel in conjunction with patient history could help to gain valuable insight into the mechanisms of drug sensitivity or resistance of tumors. These findings support further exploration of patient-derived cell lines as an ex vivo platform in cancer research and to personalize anticancer treatment.

## Supplementary information

Supplementary Information.

## Data Availability

Genomic data have been deposited in Gene Expression Omnibus (GEO) database (data accession number: GSE150963).
