# Supplementary material for: Newly established gastrointestinal cancer cell lines retain the genomic and immunophenotypic landscape of their parental cancers
Source: Sci Rep. 2020 Oct 21;10:17895. doi: 10.1038/s41598-020-74797-0 (PMC7578805; doi:10.1038/s41598-020-74797-0)
Supplement: Supplementary file 1 — Supplementary Information. [file 41598_2020_74797_MOESM1_ESM.pdf]

Supplementary Information

Newly established gastrointestinal cancer cell lines retain the genomic and immunophenotypic landscape of their parental cancers

Daniela Hirsch[1,2], Steffen Seyfried[3], Tobias Staib[3], David Fiedler[1], Christian Sauer[1], Thomas Ried[2], Stephanie Witt[4], Felix Rückert[3*], Timo Gaiser[1*]

[1]Institute of Pathology, University Medical Center Mannheim, Medical Faculty Mannheim, Heidelberg University, Germany
[2]Section of Cancer Genomics, Genetics Branch, Center for Cancer Research, National Cancer Institute, National Institutes of Health, Bethesda, MD, USA
[3]Department of Surgery, University Medical Center Mannheim, Medical Faculty Mannheim, Heidelberg University, Germany
[4]Molecular Genetic Laboratory and Biobank, Department of Genetic Epidemiology in Psychiatry, Central Institute for Mental Health (CIMH), Medical Faculty Mannheim, Heidelberg University, Mannheim, Germany
*equal contribution

Supplementary Table S1. Primary antibodies, dilutions and antigen retrieval methods used for immunohistochemistry.

**Antibody (clone)**	**Manufacturer (cat #)**	**Species**	**Dilution**	**HIAR**
CA 19-9 (1116-NS-19-9)	Dako (M3517)	mouse	1:50	none
CDX2 (DAK-CDX2)	Dako (M3636)	mouse	1:50	pH 9
CEA (II-7)	Dako (M7072)	mouse	1:200	pH 6
Chromogranin A (LK2H10)	Linaris (MAK3192)	mouse	ready-to-use	pH 6
Cyclin D1 (SP4)	DCS (CI677C01)	rabbit	1:50	pH 6
Cytokeratin 7 (OV-TL 12/30)	Dako (M7018)	mouse	1:2000	pH 9
Cytokeratin 20 (Ks20.8)	Dako (M7019)	mouse	1:200	pH 9
Cytokeratin pan (AE1/AE3)	Dako (M3515)	mouse	1:1000	pH 9
E-Cadherin (SPM471)	Fisher Scientific (MS9470)	mouse	1:50	pH 9
EMA/MUC1 (E29)	Dako (M0613)	mouse	1:200	pH 9
EpCAM (Ber-EP4)	Dako (M0804)	mouse	1:50	pH 6
HER2/neu (c-erbB2 Oncoprotein)	Dako (A0485)	rabbit	1:500	pH 6
Ki-67 (MIB-1)	Dako (M7240)	mouse	1:800	pH 6
MLH1 (ES05)	Dako (M3640)	mouse	1:25	pH 9
MSH2 (FE11)	Dako (IR085)	mouse	ready-to-use	pH 9
MSH6 (EP49)	Dako (IR086)	mouse	ready-to-use	pH 9
MUC4 (5B12)	Abcam (ab60720)	mouse	1:5000	pH 9
PMS2 (EP51)	Dako (M3647)	mouse	1:50	pH 9
SATB2 (EP281)	Medac (384R-16)	mouse	1:50	pH 9
TP53 (DO-7)	Dako (M7001)	mouse	1:50	pH 9
TTF-1 (8G7G3/1)	Dako (M3575)	mouse	1:100	pH 9
Vimentin (SP20)	Thermo Fisher Scientific (RM-9120-S)	rabbit	1:400	pH 6

HIAR, heat-induced antigen retrieval.

Supplementary Table S2. STR profiles of parental tumors and derived cell lines.

	Patient 1		Patient 2		Patient 3		Patient 4	
	**PT**	**CL**	**PT**	**CL**	**PT**	**CL**	**PT**	**CL**
**Amelogenin**	X,Y	X,Y	X,Y	X	X,Y	X,Y	X,Y	X
**CSF1PO**	10.1,12.1	12	10,12	10,12	9,10,11	11,12	11.1	11
**D5S818**	11.1,13	13	14	12	-	-	-	12,13
**D7S820**	9.3,12	9.2,12	8.3,12	8.3,12	-	9.3,10.3,13	11,12	11,12
**D13S317**	14,16	15,16	13,15	13,15,16,17	13	13	14,16	14,16
**D16S539**	11,12.1	11,12.1	11,12	11,13.1	8,9	8,14	9, 11	9,11
**TH01**	9.3	9.3	6,9.3	6,7,9.3	8,9	7,8,9	6,9.3	6, 9.3
**TPOX**	8,11	8,11	8,11	8,11	8	8,11	8,11	8,11
**vWA**	16,17	16,17	17,18	17,18	13,17	13,18	17,19	17,19

PT, parental tumor; CL, cell line.

Supplementary table S3. Mutations detected by sequencing (48 gene panel).

Sample ID	Gene	DNA sequence change	Amino acid change	Variant allele frequency
P1-PT-S1	*TP53*	c.469G>T	p.V157F	0.463
P1-PT-S2	*TP53*	c.469G>T	p.V157F	0.553
P1-CL	*TP53*	c.469G>T	p.V157F	0.779
P2-PT-S1	*KRAS*	c.38G>A	p.G13D	0.520
P2-PT-S2	*KRAS*	c.38G>A	p.G13D	0.166
P2-CL	*KRAS*	c.38G>A	p.G13D	0.493
P3-PT-S1	*BRAF*	c.1799T>A	p.V600E	0.545
P3-PT-S1	*FBXW7*	c.1394G>A	p.R465H	0.455
P3-PT-S1	*TP53*	c.902del	p.P301Qfs*44	0.250
P3-PT-S1	*TP53*	c.799C>T	p.R267W	0.200
P3-PT-S2	*BRAF*	c.1799T>A	p.V600E	0.725
P3-PT-S2	*FBXW7*	c.1394G>A	p.R465H	0.453
P3-PT-S2	*PTEN*	c.697C>T	p.R233*	0.428
P3-PT-S2	*TP53*	c.799C>T	p.R267W	0.548
P3-CL	*BRAF*	c.1799T>A	p.V600E	0.632
P3-CL	*FBXW7*	c.1394G>A	p.R465H	0.484
P3-CL	*PTEN*	c.697C>T	p.R233*	0.523
P3-CL	*TP53*	c.902del	p.P301Qfs*44	0.499
P3-CL	*TP53*	c.799C>T	p.R267W	0.463
P4-PT-S1	*KRAS*	c.35G>T	p.G12V	0.168
P4-PT-S1	*TP53*	c.476C>A	p.A159D	0.107
P4-PT-S2	*KRAS*	c.35G>T	p.G12V	0.357
P4-PT-S2	*TP53*	c.476C>A	p.A159D	0.326
P4-CL	*KRAS*	c.35G>T	p.G12V	0.661
P4-CL	*TP53*	c.476C>A	p.A159D	0.979

CL, cell line; PT, parental tumor; P1 to P4, patients 1 to 4; S1 & S2, spatially distinct samples 1 & 2.

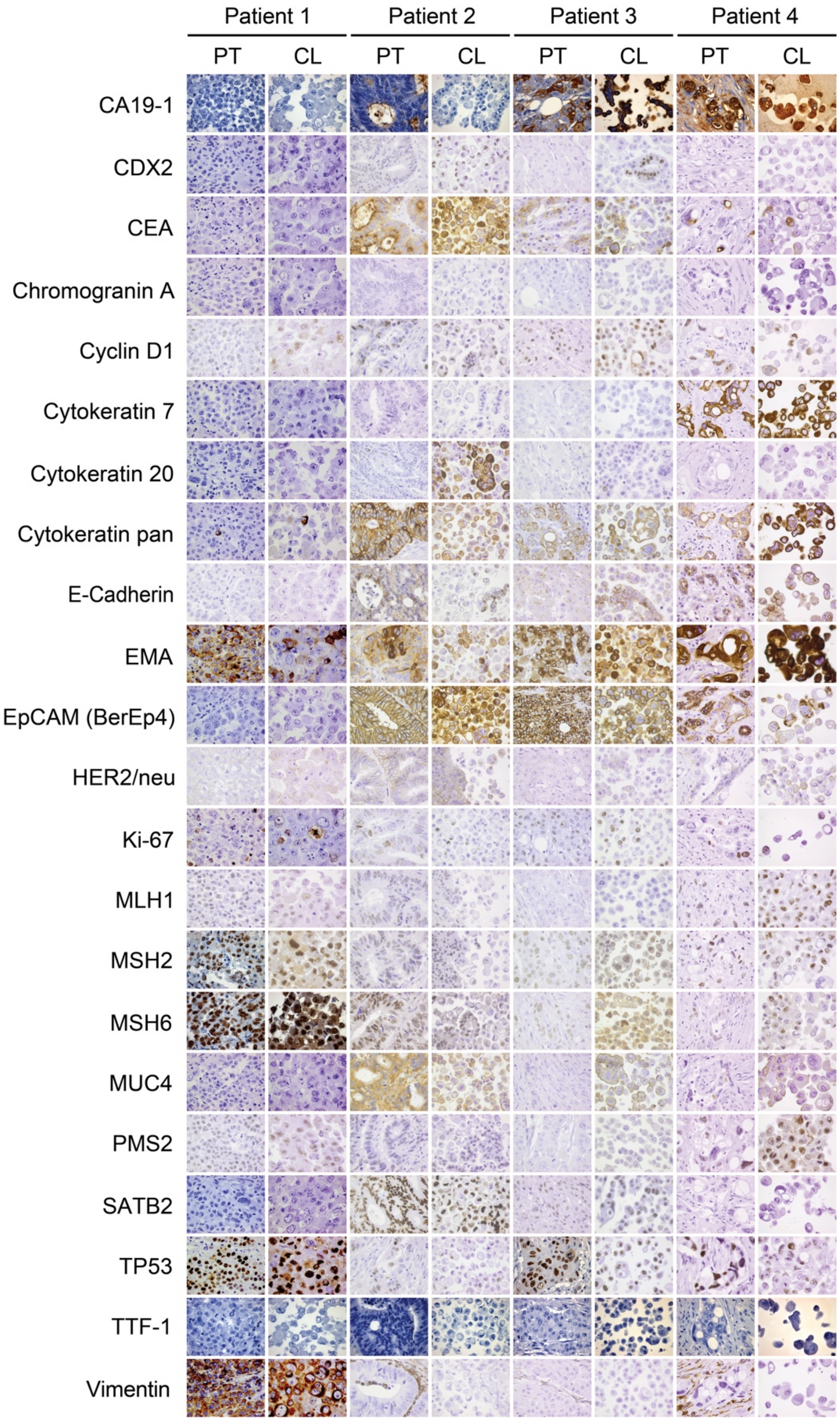

Supplementary Figure S1. Immunohistochemical staining pattern of cell lines and corresponding primary tumors, showing a high degree of similarity between cell lines and original tumors. Representative images of immunohistochemical stains for each primary tumor and respective cell line are shown.

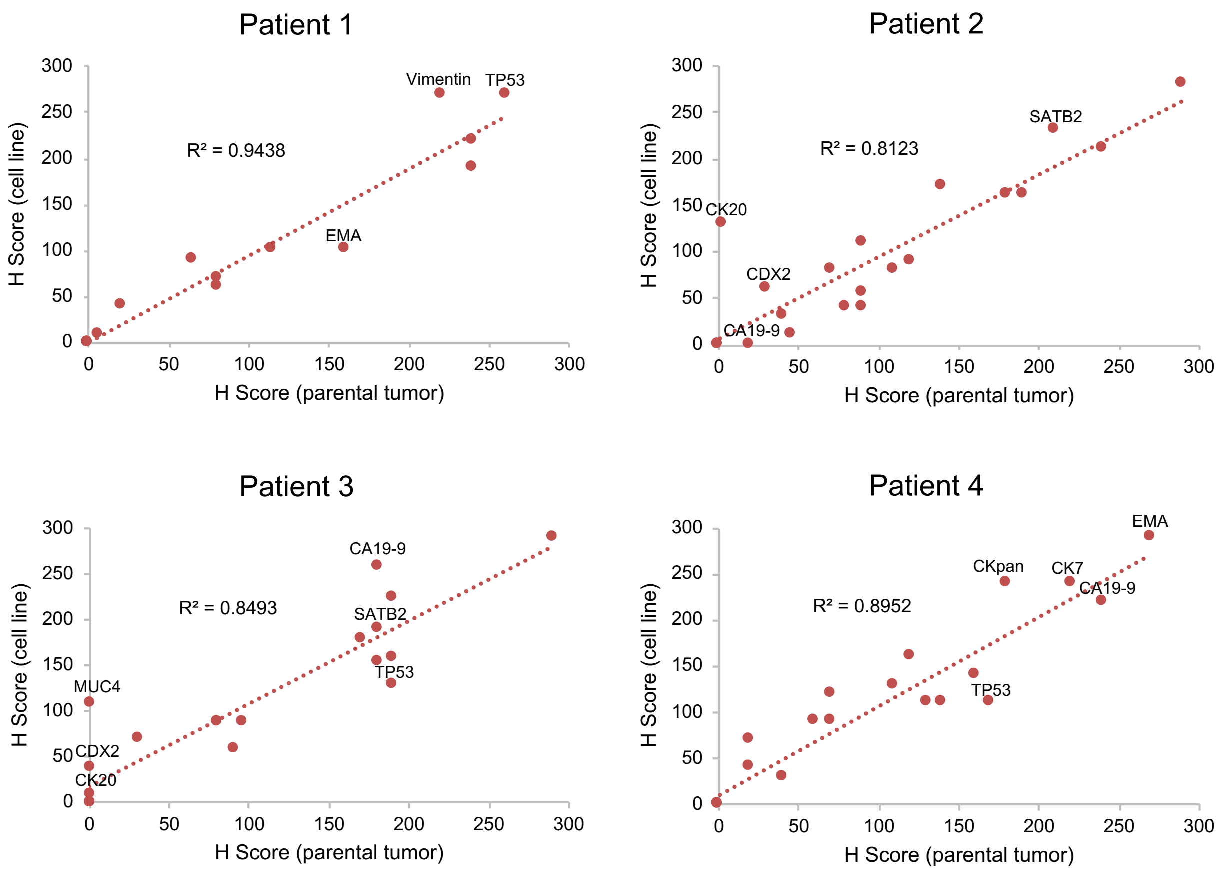

Supplementary Figure S2. Scatter plots with linear regression trendlines, depicting the relationship of H Scores of parental tumors and derived cell lines for patients 1 to 4. Selected immunophenotypic markers are highlighted. R^2, coefficient of determination (Pearson).

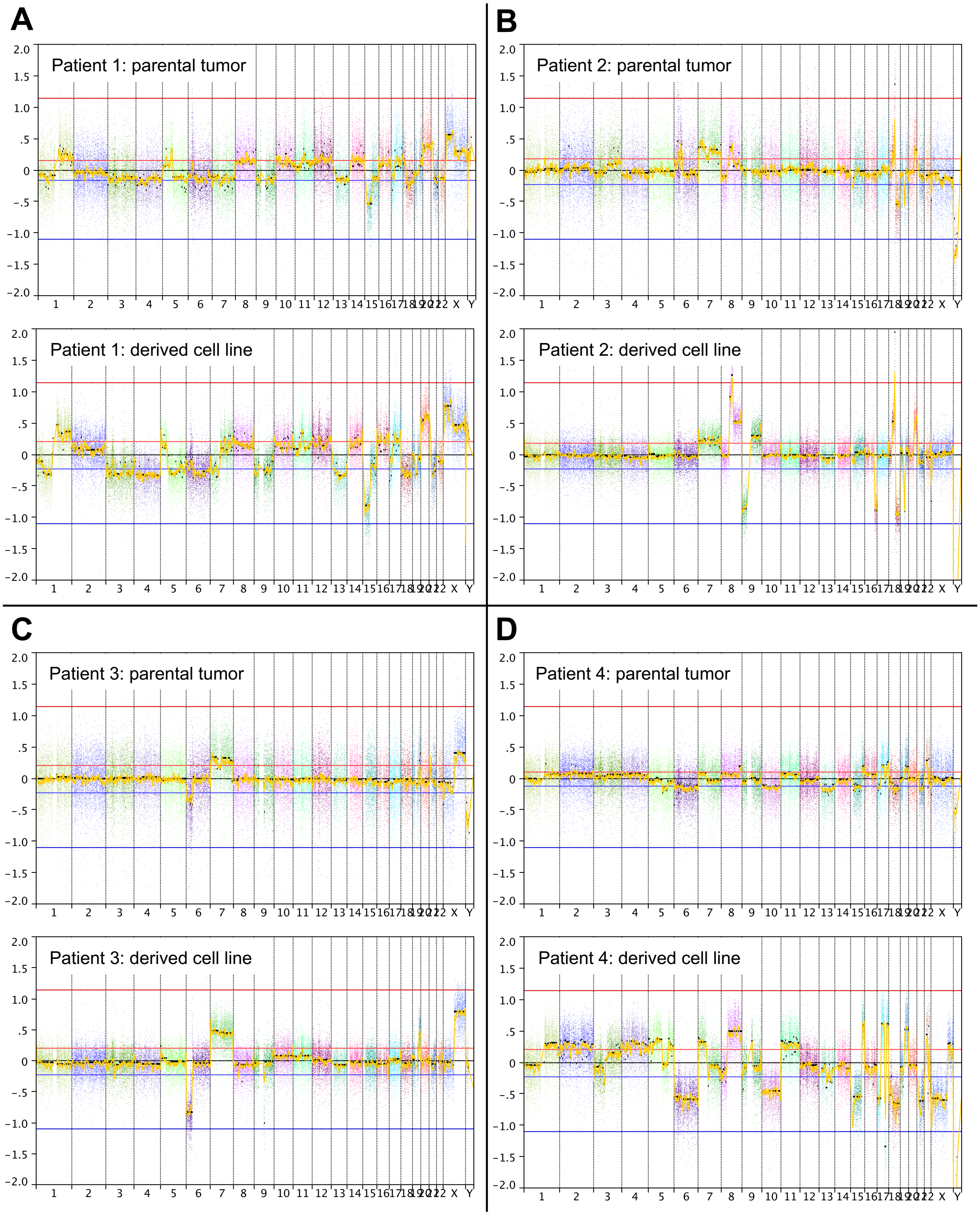

Supplementary Figure S3. Whole genome plots showing array-based comparative genomic hybridization log2 ratios (y axis) across chromosomes (x axis) per primary tumor or derived cell line sample, respectively.

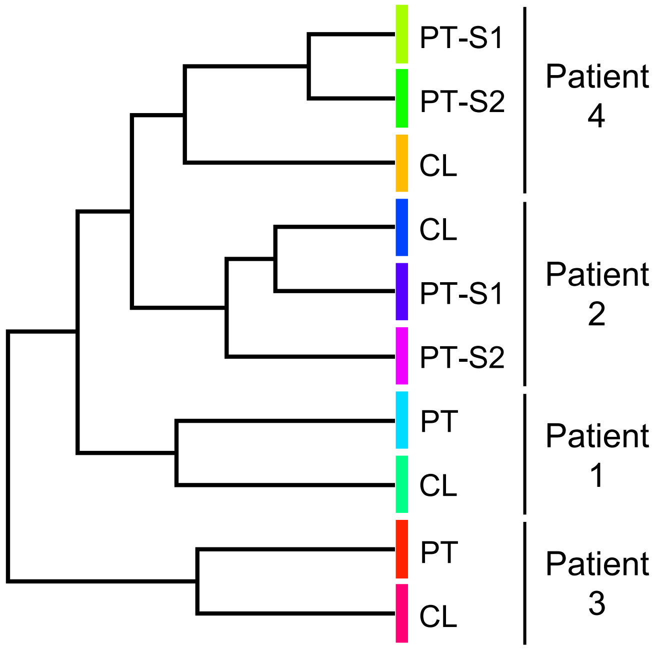

Supplementary Figure S4. Complete average linkage clustering of copy number alteration profiles of parental tumors and corresponding cell lines. From patients 2 and 4, array-based comparative genomic hybridization data from two spatially distinct samples from parental tumors were available, showing a low degree of heterogeneity at the bulk genome analysis level. These samples were included in this analysis. Reassuringly, additional samples clustered along with their corresponding cell line and other parental tumor sample. CL, cell line, PT, parental tumor, S1, sample 1, S2, sample 2.
